# Impact of a large-scale educational intervention program on venous blood specimen collection practices

**DOI:** 10.1186/1472-6963-13-463

**Published:** 2013-11-05

**Authors:** Karin Bölenius, Marie Lindkvist, Christine Brulin, Kjell Grankvist, Karin Nilsson, Johan Söderberg

**Affiliations:** 1 Department of Nursing, Umeå University, Building A, 4th floor, 901 87 Umeå, Sweden; 2 Department of Statistics, Umeå University, Umeå, Sweden; 3 Department of Public Health and Clinical Medicine, Epidemiology and Global Health, Umeå University, Umeå, Sweden; 4 Department of Medical Biosciences, Clinical Chemistry, Umeå University, Umeå, Sweden

**Keywords:** Adherence to guidelines, Education, Implementation, Intervention, Phlebotomy, Pre-analytical errors, Primary healthcare, Venous blood specimen collection

## Abstract

**Background:**

Phlebotomy performed with poor adherence to venous blood specimen collection (VBSC) guidelines jeopardizes patient safety and may lead to patient suffering and adverse events. A first questionnaire study demonstrated low compliance to VBSC guidelines, motivating an educational intervention of all phlebotomists within a county council. The aim was to evaluate the impact of a large-scale educational intervention program (EIP) on primary health care phlebotomists’ adherence to VBSC guidelines. We hypothesised that the EIP would improve phlebotomists’ VBSC practical performance.

**Methods:**

The present study comprise primary health care centres (n = 61) from two county councils in northern Sweden. The final selected study group consisted of phlebotomists divided into an intervention group (n = 84) and a corresponding control group (n = 79). Both groups responded to a validated self-reported VBSC questionnaire twice. The EIP included three parts: guideline studies, an oral presentation, and an examination. Non-parametric statistics were used for comparison within and between the groups.

**Results:**

Evaluating the EIP, we found significant improvements in the intervention group compared to the control group on self-reported questionnaire responses regarding information search (ES = 0.23-0.33, *p* < 0.001-0.003), and patient rest prior to phlebotomy (ES = 0.27, *p* = 0.004). Test request management, patient identity control, release of venous stasis, and test tube labelling had significantly improved in the intervention group but did not significantly differ from the control group (ES = 0.22- 0.49, *p* = < 0.001- 0.006). The control group showed no significant improvements at all (ES = 0–0.39, *p* = 0.016-0.961).

**Conclusions:**

The present study demonstrated several significant improvements on phlebotomists’ adherence to VBSC practices. Still, guideline adherence improvement to several crucial phlebotomy practices is needed. We cannot conclude that the improvements are solely due to the EIP and suggest future efforts to improve VBSC. The program should provide time for reflections and discussions. Furthermore, a modular structure would allow directed educational intervention based on the specific VBSC guideline flaws existing at a specific unit. Such an approach is probably more effective at improving and sustaining adherence to VBSC guidelines than an EIP containing general pre-analytical practices.

## Background

Venous blood specimen collection (VBSC) is one of the most common procedures in healthcare [1]. It is a basis for diagnosis and treatments [2,3]. VBSC is, in accordance with other healthcare practical skills, a complex procedure that demands theoretical knowledge and manual skills, as well as accuracy, ability, good caring conduct and good interaction between the phlebotomist and patient [4]. Errors in VBSC may lead to patient suffering and jeopardize patient safety [2]. Injuries related to VBSC errors are caused most often by human mistakes and relatively few are related to technical errors [5]. In addition, VBSC errors are latent and distant from direct control and thus often go unrecognized. Therefore, VBSC practices should strictly follow guidelines based on evidence and best practices [6-9].

The majority of errors within the total testing process occur in the pre-analytical phase, meaning before the sample is analysed in a laboratory [1-3,10-13]. Analytical errors (within the laboratory) and post-analytical errors (reporting and interpretation of results) are less frequent [2,13]. Some examples of the pre-analytical errors encountered are incorrect analysis ordered [14], incorrect patient identification procedures, incorrect patient preparation procedures such as insufficient patient rest, using information from outdated sources [15,16], and wrong type of collection tube [2,10]. A common reason for specimen rejection and renewed sampling is specimen haemolysis, which most often is due to incorrect specimen collection including prolonged venous stasis and not sufficiently filled tubes [1,10,17-19]. Common specimen handling errors include incorrect test tube labelling [2,15,20,21], incorrect test request management, missing tubes, and transport errors [2,8,10,14,22].

In Sweden, healthcare personnel are obliged by law to secure patient safety, keep up-to-date with healthcare guidelines, and act according to evidence-based practices [23]. Still, healthcare personnel do not always follow guidelines [14-16,21,24], and in particular are not updated with the results of new research and changed guidelines [25]. Some reasons for the low compliance to guidelines have been found to be due to personnel disagreeing with recommended guidelines and considering them unnecessary [26]. Lack of time as well as lack of support from the clinic or their superiors are other reasons [27]. Difficulties in implementation of healthcare guidelines and evidence-based care into daily practices can lead to patients not receiving the best possible care as well as being exposed to risks or adverse events [25].

Previously we used a validated self-reported questionnaire [28] and found that phlebotomists’ self-reported practical performance was poor, showing non-adherence to VBSC guidelines [14-16,21,24,29]. This motivated a large-scale educational intervention program (EIP) intending to update VBSC and implement national [7] and local [6] VBSC guidelines of VBSC personnel (2171) within the Västerbotten County Council (VLL). A large-scale EIP can be carried out by the whole healthcare organisation to improve quality of care. Few studies have evaluated the impact of large-scale EIPs on guideline adherence and healthcare practices [30]. The aim was to evaluate the impact of a large-scale EIP on primary health care phlebotomists’ adherence to VBSC guidelines. Our hypothesis was that the EIP would improve phlebotomists’ VBSC practical performance.

## Methods

### Study design and setting

A self-reported questionnaire was administered in 2007, revealing flaws in pre-analytical practices [14-17,21,29]. Because of these findings, a large-scale EIP was performed between January 2009 and November 2010, in the VLL. The controlled evaluation comprised phlebotomists from primary health care centres (PHCs) in VLL and Västernorrland’s County Council (LVN). The intervention group (IG) in this study consisted of VBSC personnel from 31 PHCs from VLL (Table 1). The control group (CG) consisted of VBSC personnel from 30 PHCs in LVN. PHCs from both county councils were located in urban as well as rural areas. Both county councils have similar working conditions and use the same national handbook [7].

**Table 1:** Numbers of IG personnel participating in the intervention over time

**Year-month**	**PHCs 1-4**	**PHCs 5-9**	**PHCs 10**	**PHCs 11-17**	**PHCs 18-24**	**PHCs 25-31**	**Total**
200909	9	-	-	8	-	-	17
200910	1	-	-	-	-	-	1
200911	5	1	-	1	-	-	7
200912	-	6	-	-	-	-	6
201001	-	5	-	-	1	-	6
201002	-	1	-	-	1	-	2
201003	-	-	-	-	2	15	17
201004	-	-	-	4	1	-	5
201005	-	-	-	-	-	4	4
201006	-	-	-	3	-	1	4
201007	-	-	-	2	5	-	7
201008	-	-	-	-	-	-	0
201009	-	-	-	-	-	-	0
201010	-	-	-	-	-	-	0
201011	-	-	1	-	-	7	8
Total	15	13	1	18	10*	27*	84

PHCs 1–4 are urban and PHCs 11–17 rural, all connected to a university hospital. PHCs 5–9 urban and PHCs 18–24 rural connected to a hospital 140 km north of the university hospital. PHC 10 is urban and PHCs 25–31 rural and connected to a hospital 130 km west of the university hospital.

* = 27/84 participated by internet link.

### Study population

**Figure 1:**
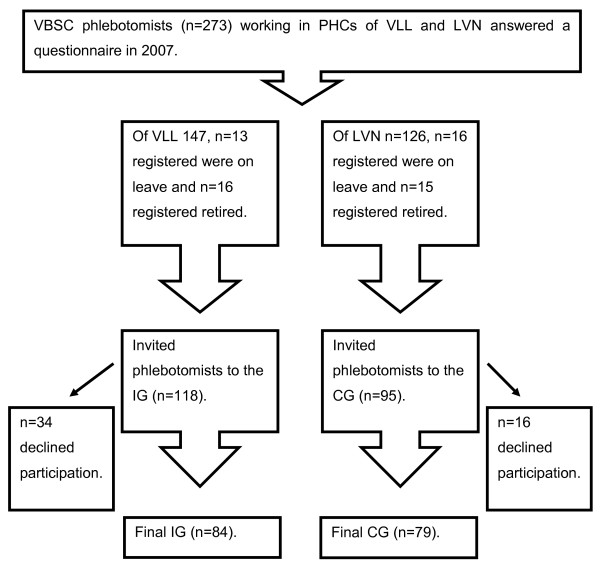
The flow chart shows the staff invited to join the study.

Inclusion criteria for both groups were set to all phlebotomists working at PHCs and having answered the questionnaire in 2007. Of 273 PHC phlebotomists, 213 were invited and 163 (77%) were finally included in the study, the IG (n = 84) and the CG (n = 79), (Figure 1). The VBSC guidelines used [6,9] were based on the national VBSC guidelines [7]. Characteristics of the study groups are summarized in Table 2.

**Table 2:** Baseline characteristics of the participants

**Background data**	**IG (n = 84)**	**CG (n = 79)**	***p***
**Sex**			
Female n	79	79	0.059*
Male n	5	0	
**Professional status**			
Registered nurses n	26	50	<0.001*
Enrolled nurses n	56	28	
Biomedical technician n	2	1	
**Age** (Year)			
Age Md (Q1; Q3)	55 (49; 60)	56 (49; 62)	0.604″
Range	28-65	38-70	
**Numbers of years employed at the job site**			
Md (Q1; Q3)	11 (7; 27)	11 (5; 20)	0.045″
Range	0-37	0-38	
**How often do you perform VBSC**			
Every day n	61	44	0.028*
Every week n	19	33	
Every month or less n	4	2	

IG = Intervention group, CG = Control group, *p =* differences at baseline between IG and CG, * = Chi-square Test, ″ = Mann–Whitney *U* Test.

### The questionnaire

The validated VBSC questionnaire [28] consisted of questions about background characteristics, patient identification, specimen collection, sample storage, information search procedures, test request management, and test-tube labelling. The questionnaires were distributed by postal service and distinguished through a coded system so the completed questionnaires from 2007 could be paired with completed questionnaires 2010–2011, 6 month after the educational intervention (Table 1). Included CG personnel completed the follow-up in April 2010. The questionnaire was completed at the individual PHC by enrolled nurses, registered nurses and biomedical technicians. The majority of the questions were answered according to a four-point ordinal scale: Never, Seldom, Often, or Always. It is noteworthy that it was pointed out clearly that the respondents were *to state how they usually performed VBSC and not how they knew how it was supposed to be performed.* Question 7c was excluded due to low reliability in the test-retest analyses [28].

### Intervention implementation strategy

The demand for a large-scale EIP (30) arose after our reports of sub-standard VBSC guideline adherence [14-16,21,24]. The VLL executive board therefore gave permission for a large-scale EIP comprising all VBSC personnel, provided it would be cheap and have minor interference with daily healthcare work (n = 2171). Given these restricted premises, laboratory instructors with experience of teaching developed a short but large-scale EIP regarding pre-analytical practices including a specific lecture of VBSC guideline practices.

The focus was on implementation of VBSC guidelines (according to the National handbook for healthcare – almost identical to the CLSI H3-A6 guidelines) [7,8] and local directives [6]. During the 2-hours lecture, emphasis was put on how to avoid haemolysis as well [19,31]. The EIP included three parts: 1) compulsory studies of the national VBSC guidelines [7] prior to education: 2) compulsory attendance at two oral lectures: 3) participants were to respond adequately to six written examination questions (randomly chosen from a bank with 24 questions) addressing education content. One of the two lectures included information of local pre-analytical errors, general VBSC practices, patient identification procedures, information search procedures, and practices important to avoid haemolysis. The second lecture addressed collection of microbiological specimens. Eight to 89 VBSC personnel participated in each lecture session. One-third of the IG (n = 27) participated through live internet link. Answers from the examination were handed over directly to the laboratory instructor, except for those who participated via a link who used postal letters to submit theirs. All participants correctly passing all examination questions received a competency certificate valid for four years.

### Analysis

VBSC questionnaires from the IG and the CG were compared between and within groups over time. Differences in change between groups were analyzed with the Mann–Whitney *U* test for ordinal data and with Chi-square test for binary data. Differences between 2007 and 2010/2011 within each group were analyzed with Wilcoxon’s signed rank test for ordinal data and with Mc Nemar’s test for binary data. To quantify the size of the difference between and within groups, standardized effect size (ES) measures were calculated [32]. Reference values for ES measures give 0.2 for a small effect, 0.5 for intermediate effect and 0.8 for a large effect [32]. If the ES is zero, the improvements are similar to the impairments (Table 3).

**Table 3:** Changes in self-reported adherence to VBSC guidelines

	**IG/CG**	**IG**				**CG**				**IG/CG**	
**Questions**	***p***^**1**^	**Md 2007/2010-2011**	**n**	***p***^**2**^	**ES**^**1**^	**Md 2007/2010-2011**	**n**	***p***^**2**^	**ES**^**1**^	***p***^**3**^	**ES**^**2**^
**Patient identification**											
7a Ask for name and identification number (4)	0.232	3.5/4	80	0.026	.18	4/4	79	0.016	.19	0.471	.06
7b I already know the patient (1)	0.380	2/1.5	79	0.019	.19	2/2	77	0.048	.16	0.028	.01
7d Control of patient’s photo-ID (4)	**<0.001**	1/2	79	**<0.001**	.34	2/2	77	0.048	.16	0.028	.18
**Collection of specimen**											
*Release stasis*											
8a -before first sample is drawn (4)	0.785	3/3	77	0.060	.15	2/3	75	0.805	.02	0.287	.09
8b -during sampling (4)	0.868	3/3	76	0.538	.05	3/3	74	0.043	.17	0.285	.09
8c -when finished sampling (1)	0.997	2/1	75	**0.006**	.22	2/2	71	1.000	0	0.040	.17
8d Keep stasis as long as necessary (1)	0.789	3/2	76	0.019	.19	3/3	73	0.886	.01	0.144	.15
*If additives*											
10a -invert the test tube immediately (4)	0.641	4/4	82	0.021	.18	4/4	78	0.063	.15	0.600	.04
10b -use automated reverser (4)	0.403	4/4	82	0.228	.09	3/4	74	0.016	.20	0.446	.06
**Information search procedures and specimen storage**											
11a Use outdated printed sampling instructions (1)	**<0.001**	2/1	78	**<0.001**	.31	1/1	73	0.415	.07	**<0.001**	.32
11b Check updated information in updated internet network (4)	**<0.001**	3.5/4	78	**<0.001**	.37	4/4	78	1.000	0	**<0.001**	.33
11d Ask colleagues for information (1)	**0.001**	3/2	81	**0.001**	.30	2/2	74	1.000	0	**0.003**	.23
11f Call the laboratory for information (1)	0.327	2/2	78	**0.007**	.21	2/2	74	1.000	0	0.046	.16
*Store test tubes*											
12a -lying on work-bench (1)	0.159	1/1	75	0.518	.05	1/1	71	0.181	.11	0.535	.05
12b -in the pocket (1)	0.307	1/1	74	0.083	.14	1/1	71	0.317	.08	0.049	.16
12c -in a test tube stand (4)	0.796	4/4	80	0.357	.07	4/4	77	0.130	.12	0.084	.14
**Test request management and test tube labelling**											
13 How often someone else marks the sampling time (5)	0.371	5/5	83	0.227	.09	5/5	79	0.443	.09	0.951	.01
15a Compare identification number with test request (4)	0.344	4/4	82	0.847	.01	4/4	79	0.132	.12	0.426	.06
15c Sign the test request (4)	0.713	4/4	82	0.152	.11	4/4	76	0.021	.19	**0.007**	.22
15d Check information if somebody else has completed it (4)	0.165	4/4	81	0.054	.15	4/4	79	0.185	.11	0.444	.06
15e Adjust sampling time if marked time differs from sampling time (4)	0.176	3/4	78	0.095	.13	4/4	76	0.519	.05	0.558	.05
15f Check that test request and test tube identification (barcode) numbers match (4)	**<0.001**	4/4	83	**0.003**	.23	4/4	77	0.961	0	0.012	.20
*Label the test tube*											
16a -before approaching the patient (1)	0.461	1/1	79	0.092	.25	1/1	71	0.567	.05	0.740	.03
16b -at patient’s side prior to sampling (4)	**0.001**	2/3	80	**<0.001**	.49	1/1	72	0.045	.17	0.060	.16
16c -at patient’s side, after sampling (4)	**<0.001**	4/3	80	0.104	.10	4/4	77	0.032	.17	0.919	.01
16d -at a later occasion (1)	0.076	1/1	79	0.052	.01	1/1	70	0.132	.13	0.019	.20
16e Somebody else labelled in advance (1)	0.017	1/1	80	0.405	.07	1/1	71	0.257	.10	0.181	.11
16f Somebody else labels after sampling (1)	0.246	1/1	80	0.096	.27	1/1	71	0.564	.05	0.173	.11
**Job satisfaction**											
19a I have enough knowledge for my daily work (3)	0.483	2/3	83	**<0.001**	.34	2/3	79	0.059	.15	0.075	.14
19b Proper VBSC is considered a priority at my PHC (3)	0.235	2/3	81	0.034	.17	3/3	78	0.760	.02	0.082	.14

*P*^*1*^ = Mann-Witney *U* Test, between IG and CG at baseline.

*P*^2^ = Wilcoxon Signed Rank Test, within IG and CG.

*P*^3^ = Mann-Witney *U* Test, for changes between IG and CG, IG = Intervention group, CG = Control group.

Significant improvement = *p* < 0.01, marked with bold font.

ES^1^ = Effect size measured by Z/n*2 square, ES^2^ = 0045ffect size measured by Z/n square.

*Md* = Median self-reported answers on a scale where either 1 or 4 is often the best possible answer. Desirable result is shown within parenthesis for each item.

Questions and items with missing answers were excluded from the analysis. IBM SPSS Statistics® (version 20), New York, United States of America, was used for all statistical analyses except for the calculation of ES, which was performed manually [32,33] by the key-author and the statistician (ML). The significance level was defined as *p* < 0.01 due to repeated tests.

### Ethics

The research plan was approved by the Regional Ethical Review Board (D-No 06-104 M). Written informed consent was obtained from the participants. All participants received verbal and written information on the study. Participants had the opportunity to decline participation in the study if they wanted, without explanation.

## Results

Evaluating the EIP, we found significant improvements in the IG regarding information search procedures, patient rest prior to phlebotomy, test request management, control of photo-identification, early release of venous stasis, and test tube labelling. The CG showed no significant improvements.

### Information search procedures and specimen storage

After the EIP, IG phlebotomists reported less use of printed, presumably outdated instructions (ES = 0.31, *p* < 0.001). They reported more use of information via the internet or the internal network, 38% reported increased use, 4% decreased use, and 58% reported unchanged procedures, (ES = 0.37, *p* < 0.001), (Table 3). The IG phlebotomists asked colleagues (ES = 0.30, *p* = 0.001) or called the clinical laboratory for help (ES = 0.21, *p* = 0.007) less often. Within the CG, no differences were found. General and in comparison, the IG phlebotomists stated improved self-reported information search procedures while the CG did not (ES = 0.23-0.33, *p* = < 0.001-0.003), (Table 3). We noted no differences in questionnaire responses regarding how to store the test tube within the IG or the CG or in comparison between the groups.

### Patient identification

After the EIP, the IG phlebotomists reported significantly improved control of the patient’s photo-identification (ES = 0.34, *p* = <0.001), (Table 3), whereas the CG did not (ES = 0.16, *p* = 0.048) (Table 3). No significant differences were found in the between-group comparison (Table 3). In the follow-up, both groups reported that they more often correctly asked for name and identification number and less often considered it to be accurate to identify by knowing the patient. No significant improvements were found within the IG or the CG, nor were there in the between comparison (Table 3).

### Specimen collection

After participating in the EIP, IG phlebotomists reported shorter venous stasis time (ES = 0.22, *p* = 0.006, (Table 3)) and more frequently allowing patient to rest before specimen collection (ES = 0.54, *p* = 0.002, (Table 4)). When comparing the IG with the CG, the IG phlebotomists reported significant improvements (ES = 0.27, *p* = 0.004) regarding patient rest (Table 4). We found no significant improvements regarding reported test tube inversion procedures within or between the groups.

**Table 4:** Changes in self-reported adherence to VBSC guidelines

	**IG/CG**	**IG**				**CG**				**IG/CG**	
**Questions**	***p***^***1***^	**2007/2010**–**2011 (%)**	**n**	***p***^**2**^	**ES**	**2007/2010**–**2011 (%)**	**n**	***p***^**2**^	**ES**	***p***^**3**^	**ES**
9 Always allow the patient to rest 11- >15 minutes prior to specimen collection	0.840	21/37	80	**0.002**	0.54	30/23	76	0.405	0.24	**0.004**	0.27
14 Always write in sampling time (0–30 minutes after sampling)	0.233	45/51	84	0.405	0.46	51/64	77	0.064	.39	0.687	0.07

*p*^*1*^ = Between IG and CG at baseline.

*p*^2^ = McNemar, (within IG and CG).

*p*^3^ = Chi-square for independence (between IG and CG), *IG* = Intervention group, *CG* = Control group.

Significant improvement = *p* < 0.01 marked with bold font. Significant improvement = *p* < 0.01 because of repeated tests. Effect size measured by Cramer’s V.

% = Percentage of correct answer.

### Test request management and test tube labelling

After the EIP, the IG phlebotomists stated that they were more accurate in checking that the test request and test tube identification number matched (ES = 0.23, *p* = 0.003) and more often labelled test tubes at the patient’s side prior to sampling (ES = 0.49, *p* = <0.001), whereas the CG had not improved significantly (Table 3). Regarding other test request management procedures, no significant differences were found within the IG, or the CG. The CG improved in the category more often sign the test request compared to the IG ES = 0.22, *p* = 0.007), (Table 3).

## Discussion

We evaluated the impact of a short but large-scale EIP on phlebotomists’ practical performance and adherence to VBSC guidelines. Our main finding was that the study demonstrated several significant improvements on IG phlebotomists’ adherence to VBSC practices. Compared to the CG we found few significant improvements. Still, guideline adherence improvement to several crucial phlebotomy practices is needed.

Staying updated with the latest laboratory sampling procedures is important since laboratory methods change, and VBSC instructions change with them [10,11]. In rural areas (as in northern Sweden), it is particularly important that healthcare personnel are aware of how to search and have access to correct information on the internal network [25]. Hence, the VBSC personnel’s ability to gain updated on-line manuals instead of outdated printed guidelines is crucial in order to maintain high levels of guideline adherence [34], probably leading to improved practical performance, more reliable test results and better patient outcomes [10,11]. In nurses’ basic education and training, the Swedish National VBSC guideline [7] which is based on for example an international guideline [8] with updated information is usually used. However, after basic education, VBSC education for phlebotomists had been absent or rudimentary until this EIP was performed.

Patient identification procedures demand accuracy as well as responsibility. High accuracy and exactness in practical skills ensure patient safety [35]. On the contrary, failure in accuracy during patient identification procedures may lead to specimen collection from the wrong patient, giving dramatic consequences with respect to diagnosis and treatment [2]. Results after the EIP show that 83% of PHCs phlebotomists often or always asked for name and identification number or checked the photo-ID compared to 70% in 2007 (*p* = 0.042) (results not shown). Still, these figures are not acceptable. One suggestion is to revise the EIP to a modular structure and have one module focusing solely on patient identification procedures, with possibilities for reflections and discussions. A study succeeded in improving blood volume in the bottles after three educational sessions indicating that a modular structure may improve performance. Long-term effects were not measured [36]. In terms of the total implementation process, it is well known that improvement of routines and practices are an on-going process. A single general EIP seldom influences all participants to improve [27].

Since the body position causes changes in plasma volume that influence the test results, patients have to rest in a sitting position before specimen collections. As test results are compared with previous results or reference intervals, samples must be taken following the same procedures [20,37]. Although personnel work more in line with guidelines it is still only 37% (Table 4) who allows the patient to rest before sampling indicating that more education is needed to sensitize the importance of rest.

In this study the IG phlebotomists reported shorter venous stasis which probably leads to more reliable test results even if we can’t prove that in this study. Prolonged venous stasis has been shown to influence test results and, for example, increase the potassium concentration [38,39]. Venous stasis is often necessary during specimen collection to localize the vein, but should be released as soon as possible or within one minute to avoid discomfort as well as affecting test results [7,8,38,39]. Lima-Oliveira *et al.* performed a phlebotomy training program [40] and eliminated a number of deviations by increasing adherence to CLSI H03-A6 guideline [8]. Thereby, a proposal was to clean the venepuncture site and let dry before stasis, in line with the Swedish national guideline [7]. If the participants cleaned the venepuncture site before stasis were not investigated in present study.

Trans illumination (cold near-infrared light-emitting diode) is also a valuable tool to localize veins especially in patients with difficult or small veins, such as children’s. A comparison between use of tourniquet and trans illumination showed no differences if using tourniquet as recommended by guidelines [38].

In contrast to Lima-Oliveira and his co-workers [40], we found no improvement in test tube inversion practices. In the Lima-Oliveira study, 17% inversed test tube correctly before training while in our study the corresponding proportion was 67% reporting that they always reversed the tube 5–10 times according to guidelines [7]. Another explanation may be that the training in our study was short (2-hours) and general. Test tube inversion after sampling is important for mixing the collected blood with additives in the test tube [8]. Test tubes are at higher risk of haemolysis if reversed more than 12 times [1,11]. Research and evidence on the importance of reversing specimens is disputed [41,42], and this may influence the phlebotomists’ adoption of guidelines [34]. A recently published study shows that vigorous mixing of test tubes does not promote laboratory variability [41]. Unclear recommendations and contradictive instructions may negatively influence the phlebotomists’ attitudes for change. There is still lack of universal consensus due to conflicting reports [43]. Motivation to change is dependent on strong evidence. If personnel believe that following guidelines would result in improved patient outcomes and improved working conditions, they probably would be more likely to change behaviours [44].

Errors related to ID or barcode linkage can cause delay or incorrect ordering of analysis, thereby delivering inaccurate test results to the patient [2,20,22]. Still, despite the intervention, 16% of the IG PHC phlebotomists reported that they sometimes labelled the test tube at a later occasion or allowed somebody else to label afterward, which is unacceptable. Internationally, frequently occurring VBSC problems, is mislabelling of test tubes; figures as high as 1 in every 165 specimens have been described [20]. In 2009, the National Board of Health and Welfare in Sweden reported 40 adverse events within blood transfusion medicine and 20 of these adverse events were due to incorrect handling or incorrect labelling of test tubes [45]. However, the VBSC guidelines are unclear on this topic. International guidelines as in The Clinical and Laboratory Standards Institute standards [8] recommend labelling test tubes whilst alongside the patient after specimen collection, and the national handbook for healthcare recommends to label the test tube before leaving the side of the patient [7] but the local directive [6] recommends labelling test tubes prior to specimen collection in accordance with the Swedish National Board of Health and Welfare [46]. Thus, it is important to standardise and clarify guidelines so they cannot be interpreted as contradictory [43]. One suggestion from a recently published editorial note is to implement bar coded specimen labels and draw blood only in the event of correct match. This would be a quite new procedure in health care and solve the problem whether blood tubes should be labelled before or after VBSC [47].

### Methodological considerations

Large-scale intervention programs are difficult to evaluate as they include a number of activities and are difficult to standardize to suit whole organizations such as all the PHCs in a specific county council [30]. Programs often have short- and long-term outcomes that have to be studied from different perspectives [30]. Even though it is difficult, we succeeded in evaluating a large-scale EIP with a validated questionnaire [28]. We also monitored low-level haemolysis in a follow-up study. Haemolysis reflects a blood specimen’s quality and is caused most often by improper specimen collection [48]. The present study has a comparative before and after design with a CG from another county council which may produce better evidence that differences are due to the programme and not to something else [30]. Thus, by using the county council as a control, we reduced the risk for spill over effect. The dropout rate was 23%, a figure that probably did not influence the overall results. Accreditation of healthcare and clinical laboratories has been increasingly utilized as a tool to try to enhance healthcare quality [49]; we used a similar approach to our completed EIP, providing all VBSC personnel who passed the exam with a competence certificate hopefully increasing the personnel’s motivation.

Both counties within this study are affected daily by different parameters that are difficult to have control over and may affect the study. Multiple components such as the lack of awareness, agreement with, or limited familiarity with guidelines [27], lack of support from the clinic or superiors, and time constraints or understaffing appear to be the main impediments for successful implementation of guidelines [27,34]. Since our EIP was mandatory, probably some were not motivated to change procedures, this could have influenced our results negatively. A weakness with the short EIP is that it contained no practical training or possibilities for deeper discussions.

The lack of more significant differences when comparing the IG to the CG may be influenced by previous questionnaire survey 2007, recall bias, the sample size, as well as a small variation among the answers (for example item 12c, 81% of the IG phlebotomists stated that they always stored the test tube in a test tube stand, the high percentage providing a small space of change). We also noted positive changes regarding items 7a and 7b (patient identification), the results implying that the VBSC personnel reflected about the procedures, already knowing that guideline adherence was low.

There was an imbalance between the CG and the IG regarding VBSC personnel professional status; more enrolled nurses were employed in the CG whereas more registered nurses were employed in the IG. However, we found no significant differences in self-reported answers regarding adherence to guidelines between those two professional groups. The frequency of VBSC showed no significant differences (*p* = 0.028) in our study.

One third of the IG performed EIP through live internet link while the others had traditional lectures; however, there were no significant differences in seven randomly selected items found between the groups. The study population was clinically relevant, as the distribution of the phlebotomists’ professional status is typical of Swedish PHC VBSC personnel.

### The clinical implication

The EIP in this study was general for pre-analytical practices. Our results indicate that regular screening of VBSC errors and an EIP could help phlebotomists to keep up to date. Yet, a revised EIP directed and compressed with increased focus on specific topics including reflections and discussions is probably more effective at improving and sustaining adherence to VBSC guidelines and practices. A “Model of Practical Skill Performance” [35] for systematic planning, useful for reflections and with focus on the specific elements in a skill together with VBSC guidelines, could probably improve and sustain guideline adherence and VBSC practices [35].

A review from 2003 graded interventions; it found that mass media campaigns, small group interactive meetings, reminders, computerized decision support, and introduction of computers to aid in practice were the most effective methods [25]. Most intervention studies have some effect, but no intervention method is good enough to obtain effective changes in all kinds of settings. It is important to study the total implementation process in order to develop interventions that are more effective in the future [25]. To develop effective EIPs, we need knowledge about pedagogical possibilities and barriers to the implementation process. E-learning is a cheap and growing educational tool, proper for large organizations and accessible to personnel in rural areas. It is also flexible and allows VBSC personnel to perform the EIP at work [50].

Implementation and translation of VBSC guidelines can be managed through a research team fostering intervention and practice assimilation by providing personal influence, motivation, retraining and instrumental assistance [51,52].

There are few evaluations of how guidelines should be effectively implemented, and it is often unclear who is responsible for implementing and sustaining guideline knowledge and practices [34]. The structure and content of the guidelines also affect their adoption; if they are easy to understand and can be tried out easily, there is a greater chance of successful implementation [27]. We suggest that VBSC guidelines should be easy and go point by point in describing their approach. Local guidelines should adapt to national and international guidelines but also secure that guidelines is in accordance with the Swedish National Board of Health and Welfare regarding VBSC.

## Conclusion

Our study demonstrated several significant improvements of the implemented educational intervention program on phlebotomists’ VBSC practical performance. Nevertheless, there are still several areas in VBSC practices that need improvement. In addition, we could not ascertain that the improvements were due solely to the EIP. We suggest future efforts to improve VBSC guideline adherence further. For instance, the educational program should provide time for reflection and discussions. Furthermore, a modular structure would allow directed educational intervention based on the specific VBSC guideline flaws existing at any specific unit. Such an approach is probably more effective at improving and sustaining adherence to VBSC guidelines than a general EIP containing pre-analytical practices for all recipients. This intervention study was, as far as we know, the first to evaluate the impact of a large-scale EIP regarding self-reported VBSC guideline adherence practices.

## Abbreviations

VBSC: Venous blood specimen collection; EIP: Educational intervention program; VLL: Västerbottens county council; PHC: Primary healthcare centre; LVN: Västernorrlands county council; IG: Intervention group; CG: Control group; ES: Effect size.

## Competing interests

The authors declare that they have no competing interests.

## Authors’ contributions

KB, JS, CB and KG proposed the original idea for the study. KB and JS collected the data and executed the statistical analyses together with ML. All the authors then discussed and participated actively in the interpretation of data continuously during the process. KB drafted the manuscript and all authors then read the draft critically, all contributed with important intellectual views to the content and approved the final draft.

## Pre-publication history

The pre-publication history for this paper can be accessed here:

http://www.biomedcentral.com/1472-6963/13/463/prepub
